# Complementing regeneration

**DOI:** 10.18632/oncotarget.4844

**Published:** 2015-07-11

**Authors:** Ali Alawieh, Aarti Narang, Stephen Tomlinson

**Affiliations:** Medical University of South Carolina, Charleston, SC, USA

**Keywords:** Immunology and Microbiology Section, Immune response, Immunity

**Figure 1:**
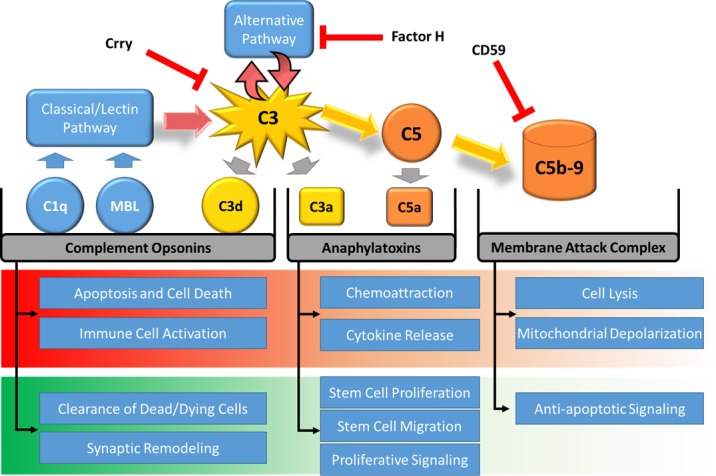
Overview of the complement cascade and its role in certain injurious and protective mechanisms, with illustration of points of inhibition by complement inhibitors mentioned in the text

Complement proteins and complement activation products with effector functions include complement opsonins (C1q, MBL, and C3d), anaphylatoxins (C3a and C5a), and the membrane attack complex (MAC). The complement system also comprises a variety of cellbound and soluble complement regulatory proteins that act on different activation pathways or at different steps in the cascade, and which prevent pathologic complement activation on normal host surfaces (Figure 1).

Complement has a well-known role in host defense, as well as pathological host cell injury when inappropriately activated, but complement is becoming increasingly implicated in a wide spectrum of additional pathophysiological mechanisms, including repair and regeneration (reviewed in [1]). Indeed, it is now clear that in some pathological conditions, complement can have a dual-edged effect and is involved in both potentiating injury as well as stimulating recovery. There is currently a great interest in the therapeutic application of complement inhibitors, but it will be important to understand the complement-dependent mechanisms involved in the balance between injury and recovery in order to apply an optimal therapeutic strategy. In recent work, we described a novel complement inhibitor that illustrates this point. It has been known for some time that complement is important for liver regeneration, and that receptor engagement by the complement activation products C3a and C5a provides an essential signal that leads to the priming of hepatocyte regeneration [2]. Liver regeneration is important for recovery after liver resection, the most common type of liver surgery, and thus inhibiting complement early in the pathway would block the generation of C3a and C5a and impair recovery. On the other hand, resection is usually performed under ischemic conditions, and it has been shown that blocking complement early in the pathway protects against ischemia reperfusion injury (IRI). We developed a complement inhibitor, CR2-CD59, that targets to sites of complement activation (via CR2 moiety) and that inhibits only the MAC, allowing generation of C3a and C5a (Figure 1) [3]. We found that when administered after hepatic ischemia and reperfusion, CR2-CD59 was as protective as CR2-Crry, an inhibitor of C3 activation, thus implicating the MAC as the prime mediator of hepatic IRI. However, in a clinically relevant model involving both ischemia/reperfusion and resection, CR2-CD59 was protective and enhanced regeneration, whereas CR2-Crry significantly worsened outcome. CR2-CD59 prevented MAC-mediated mitochondrial depolarization, and allowed a C3a/5a-mediated increase in TNF and IL-6 levels that was associated with STAT3 and Akt activation.

Regenerative responses after injury are key determinants of recovery and restoration of function. Whereas the adult liver is an exceptional organ in its capacity to regenerate, regenerative mechanisms in other organs are dependent on pluripotent cell activation in conjunction with other parenchymal or non-parenchymal stromal cells. A rapidly growing area of research is nerve regeneration and repair. There is an expanding body of evidence documenting neurogenesis in the adult central nervous system (CNS), and there is increasing interest in stimulating adult neurogenesis to promote recovery after stroke and CNS trauma. Although the mechanism underlying neurogenesis is different than that for liver regeneration, recent evidence has implicated a similarly important role for C3a and/or C5a in CNS neurogenesis after injury. Compared to the liver, however, the MAC appears to have a less prominent role in CNS injury, at least after ischemic stroke [4]. Targeting different complement pathways or different complement activation products will nonetheless likely provide strategies to minimize neurodegeneration and maximize neuroregeneration after injury. Interestingly, deficiency in C3 [5] or the lectin pathway [6] provides only acute protection against cerebral IRI, and protection is not sustained in the sub-acute phase. We have shown that the same is true when CR2-Crry (inhibits all pathways) is administered after stroke, but that the alternative pathway-specific inhibitor CR2-fH results in both acute and sub-acute protection and promotes neurogenesis (our unpublished data). Since it appears that the alternative pathway functions to amplify lectin pathway activation after stroke, this data is consistent with alternative pathway inhibition reducing, but not eliminating C3a/5a generation. Further, it has been shown that low-dose C3a receptor antagonism administered acutely after ischemic stroke is protective and causes an increase in ischemia-induced neurogenesis [6], but high dose or delayed administration impairs neurogenesis [5, 6]. It is also noteworthy that recent studies have highlighted opposing activities of C3a and C5a in certain inflammatory conditions.

A role for anaphylatoxins, and particularly for C5a, has also been demonstrated in neurogenesis after murine spinal cord injury. C5a receptor deficiency or C5a receptor antagonist treatment is protective in the acute phase after injury, but deficiency or continued antagonism causes a deterioration of outcome in the chronic phase. C5a was shown to promote astrocyte proliferation and glial scar formation via signaling through the G-protein coupled receptor C5aR1 [7].

Other complement proteins and activation products are also implicated in an interface between injury and repair. For example the cytolytic MAC can also promote survival of oligodendrocytes in a model of multiple sclerosis, and complement opsonins have been shown to play a role in synaptic pruning, as well being involved in the removal of dead and dying cells which is an important component of repair processes.

There are an increasing number of complement inhibitors becoming available that are tailored to inhibit specific pathways or activation products, and it will be important to have a detailed understanding of the dual role of complement in injury and repair for their optimal therapeutic application.
